# Angiogenesis in the degeneration of the lumbar intervertebral disc

**Published:** 2010-05-25

**Authors:** Gh David, AV Ciurea, SM Iencean, A Mohan

**Affiliations:** * ‘Regina Maria’ Military Hospital, BrasovRomania; ** ‘Bagdasar–Arseni’ Clinical Emergency Hospital, BucharestRomania; *** ‘Prof Dr N. Oblu’ Clinical Hospital, IasiRomania; **** Oradea District Clinical Emergency HospitalRomania

**Keywords:** lumbar disc hernia, angiogenesis, VEGF (vascular endothelial growth factor), FGF–2 (fibroblast growth factor 2), quality of life, risk factors

## Abstract

The goal of the study is to show the histological and biochemical changes that indicate the angiogenesis of the intervertebral disc in lumbar intervertebral disc hernia and the existence of epidemiological correlations between these changes and the risk factors of lumbar intervertebral disc hernia, as well as the patient's quality of life (QOL).

We have studied 50 patients aged between 18 and 73 years old, who have undergone lumbar intervertebral disc hernia surgery, making fibroblast growth factor and vascular endothelial growth factor level measurements, as elements in the process of appreciating the disc angiogenesis. Also, pre–surgery and post–surgery QOL has been measured, as well as the intensity of the pain syndrome.

We have identified factors capable of stimulating vascular endothelial growth (VEGF, FGF–2) for the examined disc material, but histological examination did not show angiogenesis.

The process of angiogenesis at the degenerated intervertebral disc level affects the patient's quality of life both pre and postoperatively, and may be a predictive factor for the post–operative results. Patients can prevent the appearance of angiogenesis type degenerative processes of the intervertebral disc by avoiding angiogenesis correlated factors (weight control, physical effort, and smoking).

## Introduction

At present, we notice a rise in the number of lumbar discopathy in acute, hyper–algic and paralytic forms, with a sudden debut and rapid evolution towards complications.

In the last years, studies have been crossing over to the structural and ultra structural forms (electro–microscopic) of the intervertebral disc components, in hope of discoveries with implications in the pathogenesis, prophylaxis or treatments of degenerative vertebra and intervertebral disc diseases [25].

The adult intervertebral disc is the largest non-vascular structure, the disc cells being fed by a process of remote diffusion from the pre-disc vessels. In critical nutritional or metabolic conditions there is a significant lowering of the cellular survival capacity, which explains the low structural and functional recovery of the disc tissue. A poor nutrition of the disc cells, particularly the nucleus pulpous, plays an important role in the degeneration of the intervertebral disc [3, 23, 25].

Angiogenesis is essential for the growth of the tissue and regeneration [7]. A large number of factors involved in different stages of angiogenesis have been identified–the destruction of the basal membrane surrounding the pre–existent vessels, orientation, mobilization, proliferation and the differentiation of new endothelial cells that will form the future blood vessels. Mainly, these factors are represented by:

1. Cytokines (polypeptides): VEGF (*Vascular Endothelial Growth Factor*), bFGF (*Basic Fibroblast Growth Factor*), TNF–alpha (*Tumor Necrosis Factor alpha*), TGF–alpha and –beta (*Transforming Growth Factor alpha and beta*), EGF (*Epidermal Growth Factor*), angiogenine (*Ang*), HGF (*Hepatocyte Growth Factor*), angiostatin, prostaglandins and thrombospondine.
2. Enzymes: plasmine, heparin, thrombin, hyaluronidases, collagenases, metal–proteinases, thimidinephsophorilase.
3. Extra–cellular matrix components: laminin, vitronectine, fibronectin, different types of collagen, proteoglycans, glycoseaminoglycans (GAGs).
4. Cellular surface molecules: uPAR (urokinase–type Plasminogen Activator Receptor), selectines, tissular factors, hetero–dimeric integrins [3,5,16].

Angiogenesis is a process that requires the proteolysis of the extracellular matrix, the proliferation and migration of the endothelial cells, as well as the synthesis in case of a new matrix component [12].

There are many factors capable of stimulating the growth of endothelial vascular cells (VEGF, FGF–2). Local synthesize mediators (pro–inflammatory cytokines) need long diffusion pathways to reach the nervous fibers in the periphery of the fibrous ring or the systemic blood flow. There is a recently developed hypothesis of the relationship between the neo–vascularization and neo–innervations of the intervertebral disc with degenerative modifications, but there is no clear knowledge of the actual stage of degeneration in which the neo–vascularization process emerges. The clarification of these issues is important, considering the role that neo–vascularization and neo–innervations might play in the pain simptomatology of the disc hernia. Although there is a consensus regarding the existence of blood vessels located only on the external lair of the fibrous ring of the normal adult intervertebral disc, there is a continuous deviate as far as the existence of a vascular growth process on the internal lair of the fibrous ring is concerned, during the degeneration process.

In this study, the neo–vascularization has been investigated on herniated disc material that has undergone surgical extirpation, but the vascular growth in the interior of the fibrous ring remains difficult to appreciate [15,18].

## Material and methods

We have made a longitudinal, analytical, cohort study, of ambispective type, through which we can show the hysto–biochemical aspects of the degenerative pathology of intervertebral discs, correlated with clinical aspects, risk factors, the results of pre–operatory investigations and the patient's quality of life before and after the surgical procedure. 

The target population of this study is represented by intervertebral disc hernia patients who were hospitalized in the neurosurgery clinic of ‘Regina Maria’ Military Emergency Hospital in Brasov, during 01.01.2006–30.06.2009.

We have studied the impact of the disc degenerative process (angiogenesis process) on the pre and post–operatory quality of life, the alteration of the health–state parameters (pain, mobility, usual activities, depression, self–care), on 50 patients who took part in the study during 01.01.2006–30.06.2009.

The study lot was established by selecting the cases clinically diagnosed with lumbar intervertebral disc hernia, confirmed by imagistic investigations, MRI or CT. Out of 2,744 cases admitted with lumbar disc hernia, 1,366 patients have required surgical intervention. Out of 1,366 patients, we have randomly selected 125 subjects during 01.03.2009–31.05.2009, for the hystochemical study of the extirpated disc fragments, out of which 50 have met the requirements to be included in the study.

There have been 35 (70%) men and 15 (30%) women in the analyzed series. Patient age was between 10 and 73 years old, the average age being 45.76 years old. The average age of men was of 45.17 years old while the average age of women was of 47.13 years old.

Patient age repartition is relevant, showing the high frequency of the disease in the active groups of population, presenting a great social efficiency.

Professional categories repartition was the following: 34% (16 cases with high physical requirements) industry workers, agriculture workers, plumbers; 6% drivers (3 cases in which the sitting position sets high tension on the last two lumbar vertebras, with implications in the apparition of discopathy at this level), engineers, administrators, teachers, medical nurses and 40% retired or unemployed.

Out of the study lot, 50% of the patients had L5 hernia, 46% had L4 hernia and 4% L3.

Clinical data analysis have shown that 80% of the patients have presented with sciatic accompanied or not by lumbalgia, 52% – mobility disorders, 68%–reflex disorders, 100%–radiculary sensitivity disorders, 100%–positive elongation signs and 2% Lasegue controlateral sigh, without sphincter disorders.

Lumbar intervertebral disc hernia diagnosis has been established by anamnesis, neurological and neuro–imagistic exploration, and was surgically confirmed.

Anamnesis has shown pain triggering factors, relationship of pain with physical effort or professional specificity, pain evolution, the influence of postural changes and of medication on sciatic, as well as the degree of the patient's life quality degeneration.

The evaluation of the patient's life quality has been made via the Euro Quality of Life 5D scale (EQ–5D). 

The Euro Quality of Life 5 Dimensions (EQ–5D) has two parts: a descriptive part that is a self–evaluation scale based on five criteria: mobility, self–care, daily activities, pain/discomfort, depression and three levels on each criterion (no problems, moderate problems, severe problems). The quotient named EQ–5D index is calculated based on the answers, and there are 243 possible health–states. The second part is an analogue–visual vertical scale graded 0 to 100, where 0 is the worst state of health imaginable and 100 the best. The second part scale is also named the ‘thermometer of health state’. The evaluation was made pre–operatory and three months post–operatory.

The disc materials were obtained during the surgical act and were fixated on paraffin. Histopathological studies on the 50 selected patients were made by using a usual coloration (hematoxiline–eosin) and special colorations (PAS, Giemsa and Van Gieson).

Imunohistochemical techniques (IHC) have been made on section 3 µm thick, obtained from the paraffin blocks that include tissue fixated with 10% formaldehyde solution. The technique is bi–stadial, indirect, made by a polymer based system of detection (En Vision+ Dual Link System–HRP, Dako, Carpinteria, CA). Having the goal of insuring a high standard degree of quality of the study, we have made an internal IHC control technique by implementing and certificating quality insurance (ISO 9001/200). All the tests were counter–colored with Meyer hematoxiline, examined and photographed with a Nikon Eclipse 600 microscope.

For the study of the angiogenesis process at disc levels we have determined:

- FGF–2 factor–a member of the growth family factors that stimulates proliferation of epithelial cells and of mesenchymal and neuroectodermal origin
- VEGF, part of the growth factors with angiogenesis activity. VEGF is a dimer glycoprotein with a structure close to PDGF (platelet derived growth factor)

## Results

### Histochemical results

Histological examination has not shown a neo–vascularization process in extirpated disc fragments.

We have identified some factors capable of stimulating endothelial vascular cell growth (VEGF, FGF–2) on the examined disc materials.

**Figure 1:**
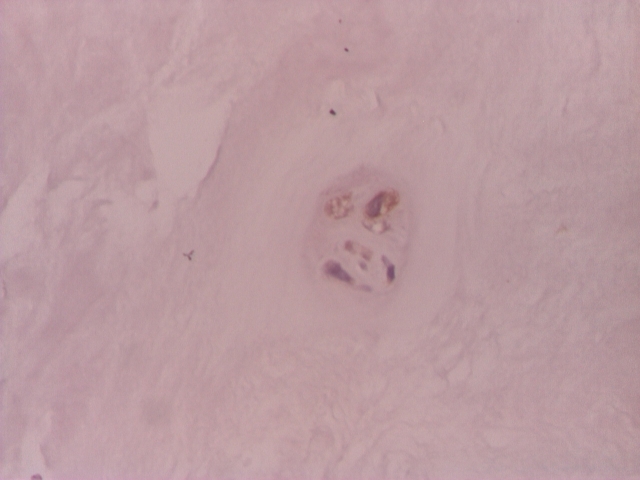
Intervertebral disc: positive VEGF chondrocyte–like cells– IHC 200x

**Figure 2:**
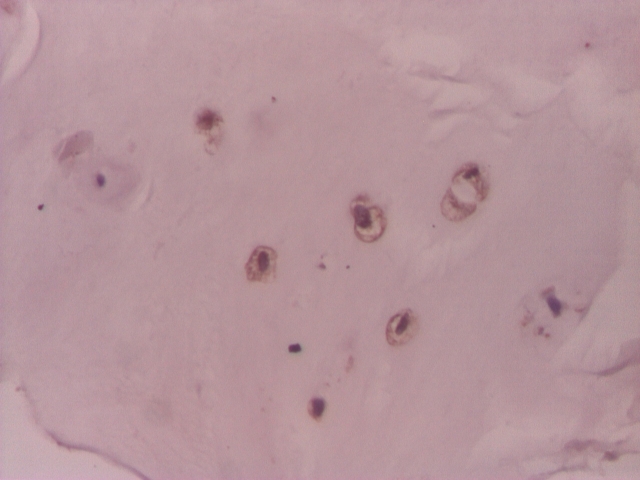
Intervertebral disc: VEGF in chondrocyte–like cells– IHC 200x

**Figure 3:**
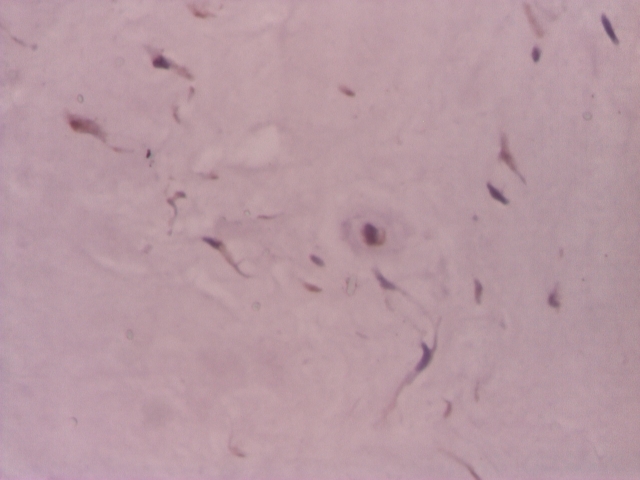
Intervertebral disc, VEGF positive in rare chondrocyte–like cells and in fibroblast–like cells–IHC 200x

**Figure 4:**
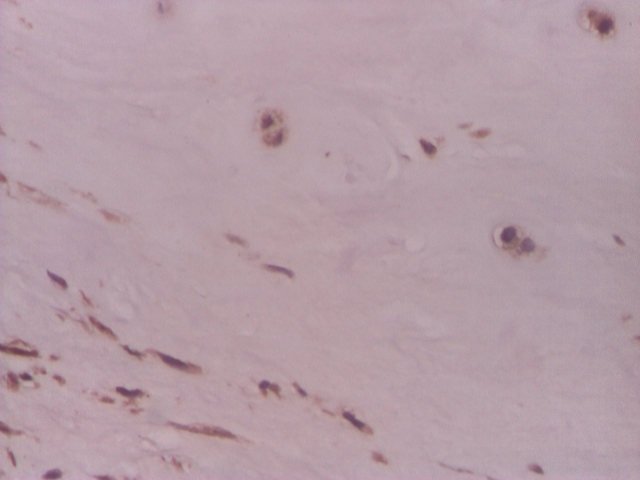
Intervertebral disc, VEGF relatively rare in chondrocyte–like and fibroblast–like cells – IHC 200x

VEGF was positive in chondrocyte–like cells in 31(62%) of the cases of the lot. In 4 cases (8%) VGEF was positive both in fibroblast–like cells and chondrocyte–like cells. In 19 (38%) cases of the lot, VEGF was negative (Figure 1, Figure 2, Figure 3, Figure 4).

**Figure 5:**
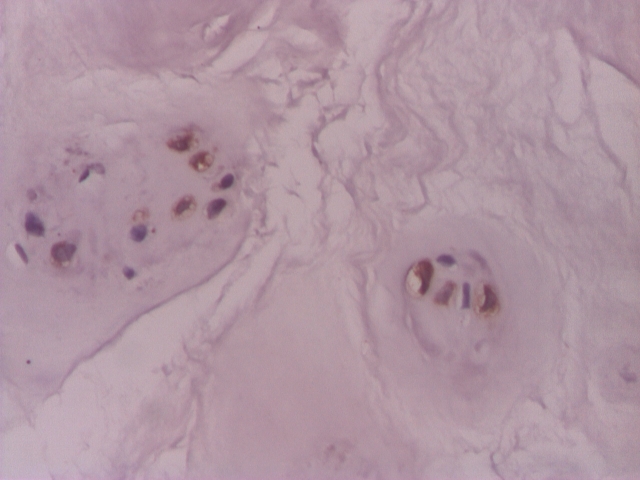
Intervertebral disc, positive FGF–2 in chondrocyte–like cells IHC 200x

**Figure 6:**
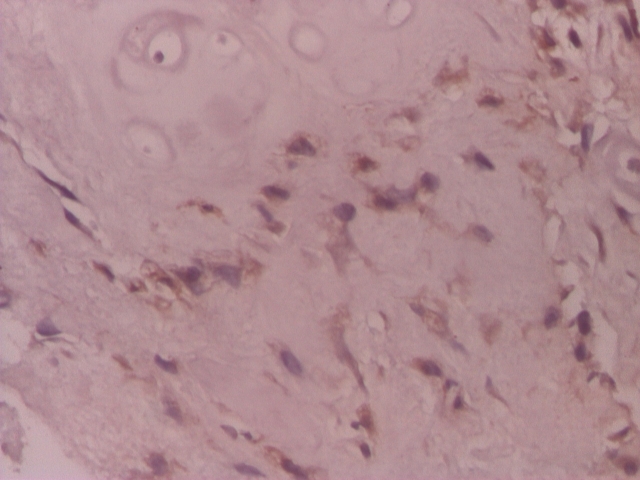
Intervertebral disc, FGF–2 positive in chondrocyte–like and fibroblast–like cells IHC 200x

FGF–3 reaction in the study lot was positive in 49 (98%) cases in chondrocyte–like cells, and, in 7 (14%) cases the reaction was positive both in chondrocyte–like and fibroblast–like cells. In only one case (2%), the reaction was negative. (Figure 5,Figure 6)

**Table 1:** Results of VEGF tests

VEGF	Number of cases	Percentage
neg	19	38.0%
pos chondrocyte–like	8	16.0%
pos chondrocyte–like and fibrobl–like	3	6.0%
pos very rare chondrocyte–like	5	10.0%
pos rare chondrocyte–like	14	28.0%
pos rare chondrocyte–like and fibrobl–like	1	2.0%
Total	50	100.0%

**Table 2:** Results of FGF–2 tests

FGF–2	Number of cases	Percentage
neg	1	2.0%
pos chondrocyte–like	21	42.0%
pos chondrocyte–like and fibroblast–like	7	14.0%
pos very rare chondrocyte–like	1	2.0%
pos rare chondrocyte–like	20	40.0%
Total	50	100,0%

### Quality of life evaluations

**Graph 1:**
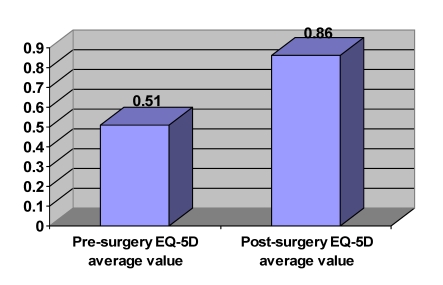
Pre and post–surgery EQ–5D average values

**Graph 2:**
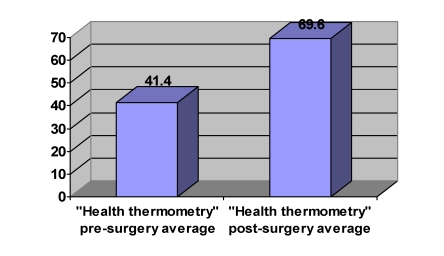
Pre and post–surgery ‘health thermometry’ average

The EQ–5D pre–surgery index was of 0.51 (standard deviation –SD=±0,2) and the  ‘health thermometry’ was of 41,4 (SD=±12,2). This data indicates that patients had a high degree of disability and alteration of the quality of life in the pre–surgery period. (Graph 1 and Graph 2).

Post–surgery data after 3 months suggest that the surgical intervention is an efficient therapeutic approach of intervertebral disc hernias (P<0.05). Therefore, EQ–5D index has improved to 40.7% (by 0.35) (SD=±0,09), and after the ‘thermometry of health’ the improvement was of 40.5% (by 28.2) (SD=±9,6).

Neo–angiogenesis process analysis in post–surgery pain evolution shows that there is a 1.53 higher risk of postoperative pain after 3 months, on patients with positive VGEF. The effect is important both from the clinical and statistical points of interest. (Table 3).

**Table 3:** Correlation between pain (EQ–5D criteria) and vascular endothelial growth stimulating factors (VEGF, FGF–2)

	Post–surgery pain +	Post–surgery pain –	
VEGF +	25	6	(31)
VEGF –	10	9	(19)
	(35)	(15)	50

RR= 1,53 (0,97<RR<2,43) for a trust interval of  95%, X^2^=4,4; correlation quotient. r^2^0,25.RA=0,28.

The mass of those with histopathological modification of positive VEGF and post–surgery pain sort is 3 times higher than the ones of those with negative VEGF results (73% vs 27%)

**Table 4:** Average EQ–5D index on pre–surgery on patients with or without angiogenesis phenomena

VEGF	Number of cases	Total score	Average EQ–5D
Neg	19	10.8489	0.5710
Pos. chondrocyte–like	8	4.1623	0.5324
Pos. chondrocyte–like and fibroblast–like	3	1.9338	0.6446
Pos. very rare chondrocyte–like	5	2.0206	0.4041
Pos. rare chondrocyte–like	14	6.4281	0.4695
Pos. rare chondrocyte–like and fibroblast–like	1	0.4994	0.4994

## Discussions

In the process of intervertebral disc degeneration the nutrient offer is blocked; with less blood flowing through the vertebra, there is a lowering in the available nutrients quantity, a lowering of the pH levels and implicitly cellular death. (Kauppila, L et al. Spine 22:1642-1649, 1997; Kurunlahit, M et al. Radiology 221:779-786, 2001). In addition to the narrowing of the major lumbar blood vessels, many studies have shown a decrease in the blood–flow in the interior of the vertebral body, which is the reason for the loss of nutrients and the degenerative disease of the disc, added to the negative effects of nicotine and the aging of the disc. (Iwahahi, M et al. Spine 27:1396-1401, 2002; Boos, N et al. Spine: 27:2631-2644, 2002). 

Angiogenesis is essential in the growth of tissues and in the regeneration process. There are many factors that are capable of stimulating the growth of endothelial vascular cells, including the vascular endothelial growth factor (VEGF).

In this study we have concluded that VEGF was positive in chondrocyte–like cells in 62% of the cases and, in 8% of the work–lot, VEGF was positive on both fibroblast–like and chondrocyte–like cells. FGF–2 reaction was positive in 98% of the cases in chondrocyte–like cells, and in 14% of the cases in both types of cells. Only one case had a negative reaction. 

Therefore, the capillaries in the herniated disc tissue are newly–formed and the results have shown that FGF–2 and VEGF are participants in the neo–vascularization process. The presence of FGF–2 in fibroblasts suggests that this growth factor restores the function of these cells, and eventually, the proliferation of cells and the production of extracellular matrix components. In addition, numerous studies, consolidating the MRI results, suggest that the disc resorption is more frequent when the epidural space is completely exposed and this aspect is correlated with the degree of vascularization. (9) By means of histopathological examination we have observed a marked macrophage infiltration and neo–vascularization.

The angiogenesis factor, VEGF, might be involved in the neo–vascularization of the tissue in lumbar intervertebral disc hernia, because we have proved that VEGF and FGF–2 are present on the examined disc material.

The analysis of the angiogenesis process in the evolution of post–surgery pain shows that there is a 1.53 higher risk of patients with positive VEGF to present post–operatory pain after 3 months. The observed effect is important both from the clinical and statistical points of interest [RR= 1,53 (0,97<RR<2,43 for a 95%, X^2^=4,4 trust interval].

The impact of co–morbidities on the angiogenesis process indicates a higher percentage of positive VEGF results in patients with no associated pathology 68% versus 56% VEGF positive with co–morbidities. The effect is clinically important but has no statistical value (for a trust interval of 95%, X^2^=0,76), we can therefore set this on behalf of the degeneration of the intervertebral disc and not on behalf of other associated pathologies that might induce an angiogenesis process.

The angiogenesis process influences the intensity of the pain manifested in intervertebral disc hernias. The risk of those who present with a hyper–algic VEGF positive form is x2.45 higher than those without pre–surgery pain. There is a clinical and statistical importance in this result (RR= 2,45 (0,95<RR<6,25) for a trust interval of 95%, X^2^=4,58.

The percentage of those with angiogenesis and hyper–algic modification in pre–surgery is relatively higher than the percentage of those with negative VEGF histopathological results (80% versus 20%). We can appreciate that the prevention of neo–angiogenesis would eliminate 30% of the hyper–algic forms of hernia (RA=0.3)

The study shows that 96% of those with positive VEFG have improved their self–care capacity after the surgical intervention, but there is also a 4% quotient that have not improved this parameter, compared to those with negative VEGF histopathological results and had a postoperative improvement of 100%.

Angiogenesis processes do influence the postoperative improvement of daily activities of the intervertebral disc hernia patient, but with no statistic significance (X^2^=3,41).

The present study shows that 83.8% of those with positive VEGF have improved the daily activity capacity after the surgical procedure, but there is a percentage of 16% who have not improved this parameter compared to those who were VEGF negative and had a postoperative improvement of 100%.

Angiogenesis processes negatively influence the improvement of postoperative mobility for 3 months after the surgery in patients with intervertebral disc hernias (X^2^=4,18), only 83.87% of those with angiogenesis processes have presented an improvement in postoperative mobility.

The risk of no improvement in the anxiety state at 3 months after surgery in patients with positive VEGF is x2.15 greater than the one of those with negative VEGF. Only 22.5% of those with positive histopathological VEGF have declared having no postoperative depressions, compared to the 89.5% VEGF negative and improved psychic wellbeing. [X^2^=1,16, RR=2,15 (0,5<RR<9,27)].

Postoperative ‘health thermometry’ indicates a strong correlation between the presence of the angiogenesis process at the intervertebral disc level, and a dissatisfactory improvement in the quality of life [ X^2^=10,96; RR=2,82 (1,29<RR<6,15)].

The risk of those with positive VEGF positive histopathological results for no improvement in the quality of life (a score under 70) is 2.82 greater than that of those without angiogenesis process; only 25.8 of those with positive VEGF have reached a postoperative score above 70.

Similarly, the angiogenesis process of the intervertebral disc significantly influences both clinically and statistically the patient's quality of life in pre–surgery stage (average EQ–5D pre–surgery score with positive VEGF is 0.48 and that of those with negative VEGF is 0.57 (X^2^=4,64) (Table 4).

There is a strong correlation between smoking and angiogenesis at the intervertebral disc levels (X^2^=4,64 ). The risk to present positive VEGF in smokers is x1.62 higher than the risk of non-smokers. The percentage of the new–vessel formation process is higher in smokers than the one in non–smokers (85.71% vs. 52.77% in non–smokers) [RR= 1,62 (1,12<RR<2,36) for a confidence interval of 95%, X^2^=4,64].

Excess weight also influences angiogenesis at the level of the intervertebral disc. The risk to present positive VEGF in overweighed patients is x1.43 higher than the one of normal–weight patients. The effect, although with no statistical significance has an important clinical impact (X^2^=2,4). The percentage of angiogenesis process in obese patients is higher than the one of normal–weight patients (71,4% in obese versus 50% in normal–weight).  [RR= 1,43 (0,88<RR<2,31) for a confidence interval of 95%, X^2^=2,4]. 

The risk of having positive VEGF in sedentary patients is x1.33 higher than the one of those with an active lifestyle. The studied effect, although with no significant statistical value (X^2^=1,19), resulted into differences which have a major clinical value. The percentage on neo-angiogenesis in sedentary patients is higher than that of those with a healthy active lifestyle (66.6% vs. 50%) [RR= 1,33 (1,75<RR<2,86) for a confidence interval of 95%, X^2^=1,19]

Our data analysis shows a strong association between the effort risk factor and the angiogenesis process at level of the intervertebral disc. (X^2^=4.13). The risk to present positive VEGF results in those who overwork the back muscles is x1.83 higher than the one of those who do not cross the maximum limit of bio–mechanical solicitation. The percentage of angiogenesis in those that overwork back muscles is higher than the one of those who do not make excessive effort (83,8% versus 57,87%).  [RR= 1,83 (0,89<RR<3,75) for a confidence rating of 95%, X^2^=4,13]

The study shows that the presence of FGF–2 in condrocite–like cells and in fibroblast–like cells has no statistical relevance on postoperative pain ( X^2^=0,48), although in 67.34% of the patients' lot presenting positive FGF–2, postoperative pain was accounted for.

**Graph 3:**
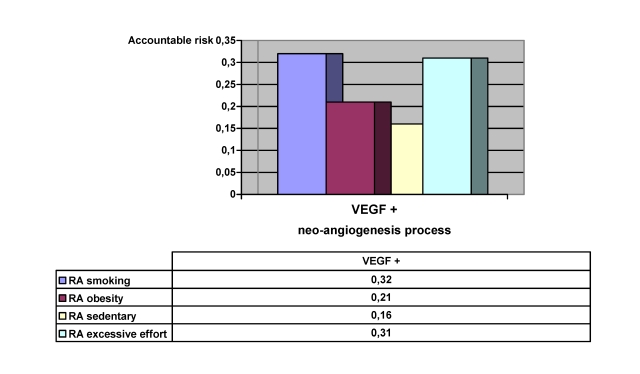
Accountable risks that can be avoided in the neo–angiogenesis process

The risk accountable for the relative risk characterizes both the aggressiveness of the factor and the frequency of that action, and, therefore, it serves in identifying those problems that can be addressed and solved with maximum efficiency and an optimal use of available resources. By eliminating smoking, we would reduce angiogenesis processes by 32%; with an adequate control of body weight we would eliminate 21%; by therapy and exercise that strengthen the abdominal and para–vertebral musculature we would eliminate 16% and by eliminating excessive effort we would reduce new vessel formation by 31%. (Graph 3).

The study also followed the determination of the risk factor's impact on the cellular biology of the degenerated intervertebral disc. There are risk factors that cannot be avoided in the disc hernia: age, gender, history of lumbar trauma, disc hernias or lumbar surgery. The aging process of lumbar intervertebral discs, as well as frequent injuries of the discs or spinal muscles, raises the predisposition to back pains.

There are risk factors that can be avoided: professional issues or other activities that raise the risk of disc herniation (lifting heavy objects, twisting or bending, hard physical exercises etc.), smoking and obesity.

Some patients may prevent the debut of degenerative processes of the intervertebral disc, or the aggravation of the symptoms by avoiding these risk factors, if they adopt a correct posture, avoiding intense physical efforts or if they stop smoking and avoid aggravating factors (moisture, low temperatures).

## Conclusions

1. The angiogenesis process which influences the intensity of the pain in intervertebral disc hernias has a negative impact on the postoperative pain improvement, mobility and overall quality of life. The improvement of postoperative depression and self–care capacity of the patient, and the postoperative improvement of daily activities are affected by these histopathological modifications.
2. We consider that the angiogenesis factor, vascular endothelial growth factor, is involved in the neo–vascularization of tissues in lumbar disc hernias. The study demonstrates that VEGF and FGF–2 are present in the herniated disc material.
3. The angiogenesis process of the intervertebral disc affects the pre–surgery quality of life (average EQ–5D pre–surgery on patients with positive VEGF is 0.48, and those with negative VEGF have a score of 0.57 (X^2^=4.64). We can appreciate that preventing neo–angiogenesis would eliminate 30% of the hyper–algic forms of hernia (RA=0,3).
4. The study shows that there is a strong epidemiological association between smoking, excessive effort and the angiogenesis process. Obesity and sedentary way of life raise the percentage of the new blood vessel formation processes.
5. By eliminating smoking, we would reduce angiogenesis processes by 32%; with an adequate control of body weight we would eliminate 21%; by therapy and exercise that strengthen the abdominal and para–vertebral musculature we would eliminate 16% and by eliminating excessive effort we would reduce new vessel formation by 31%.

## References

[1] Avram E, Ciurea AV, Ciubotaru VG (1999). Managementul modern în organizaţiile sănătăţii– perspective in serviciile de neurochirurgie.

[2] Colombini A, Lombardi G, Corsi MM (2008). Pathophysiology of the human intervertebral disc. Int J Biochem Cell Biol.

[3] Crock HV, Goldwasser M, Yoshizawa H (1991). Vascular anatomy related to the intervertebral disc. Biology of the Intervertebral Disc.

[4] Deyo RA, Bass JE (1989). Lifestyle and Low–Back Pain: the influence of smoking and obesity. Spine.

[5] Eyre DR, Wu JJ, Fernandes RJ, Pietka TA (2001). ARecent developments in cartilage research: matrix biology of the collagen heterofibril network. Biochem Soc Trans..

[6] Fahmy RG, Dass CR, Sun LQ, Chesterman CN, Khachigian LM (2003). Transcription factor Egr–1 supports FGF–dependent angiogenesis during neovascularization and tumor growth. Nat Med.

[7] Folkman J (2003). Fundamental concepts of the angiogenic process. Curr Mol Med..

[8] Hall RA, Kang JD (2000). Degeneration, repair, and regeneration of the intervertebral disc. Curr Opinion Rheum.

[9] Haro H, Kato T, Komori H, Osada M (2002). Vascular endothelial growth factor (VEGF)–induced angiogenesis in herniated disc resorption. . J Orthop Res.

[10] Heliovaara M (1989). Risk factors for low back pain and sciatica. Ann Med..

[11] Hicklin DJ, Ellis LM (2005). Role of the vascular endothelial growth factor pathway in tumor growth and angiogenesis. J Clin Oncol..

[12] Johnson WE, Evans H, Menage J, Eisenstein SM, El Haj A, Roberts S (2001). Immunohistochemical detection of Schwann cells in innervated and vascularized human intervertebral discs. Spine.

[13] Kara SA, Toppare MF, Avsar  F (1999). Predictors of early improvement in low back pain amongst consulters to general practice: the influence of pre–morbid and episode–related factors. Pain.

[14] Kim DJ, Moon SH, Kim H (2003). Bone morphogenetic protein–2 facilitates expression of chondrogenic, not osteogenic, phenotype of human intervertebral disc cells. Spine.

[15] Leung VY, Chan D, Cheung  KM (2006). Regeneration of intervertebral disc by mesenchymal stem cells: potentials, limitations, and future direction. Eur Spine J.

[16] Lipson SJ, Muir H (1981). Experimental intervertebral disc degeneration: morphologic and proteoglycan changes over time. Arthritis Rheum.

[17] Moon SH, Nishida K, Gilbertson LG (2002). Responsiveness of human intervertebral disc cells to adenovirus mediated transfer of TGF–1 cDNA in 2D and 3D culture systems: comparison to exogenous TGF–1. Presented at the International Society for the Study of the Lumbar Spine meeting.

[18] Nisato RE, Tille JC, Jonczyk A (2003). Alphav beta 3 and alphav beta 5 integrin antagonists inhibit angiogenesis in vitro. Angiogenesis.

[19] Raj PP (2008). Intervertebral disc: anatomy–physiology–pathophysiology-treatment. Pain Pract.

[20] Roberts S, Eisenstein SM, Menage J, Evans EH (1995). Mechanoreceptors in intervertebral discs. Morphology, distribution, and neuropeptides. Spine.

[21] Roberts S, Evans EH, Kletsas D (2006). Senescence in human intervertebral discs. Eur Spine J.

[22] Roberts S, Menage J, Urban JPG (1989). Biochemical and structural properties of the cartilage end–plate and its relation to the intervertebral disc. Spine.

[23] Trout JJ, Buckwalter JA, Moore KC (1982). Ultrastructure of the human intervertebral disc: Ⅱ. Cells of the nucleus pulposus. Anat Rec.

[24] Videman T, Battie MC (1999). The influence of occupation on lumbar degeneration. Spine.

[25] Zhao CQ, Wang LM, Jiang LS (2007). The cell biology of intervertebral disc aging and degeneration. Ageing Res Rev.

